# Supplementary material for: COVID-19 infection: Disease detection and mobile technology
Source: PeerJ. 2020 Nov 9;8:e10345. doi: 10.7717/peerj.10345 (PMC7659622; doi:10.7717/peerj.10345)
Supplement: Supplemental Information 1 [file peerj-08-10345-s001.docx]

**^#^List of Abbreviations:**

| Country Territory Code | Countries and Territories |
| --- | --- |
| USA | United States of America |
| ITA | Italy |
| FRA | France |
| ESP | Spain |
| GBR | United Kingdom |
| BEL | Belgium |
| DEU | Germany |
| IRN | Iran |
| CHN | China |
| BRA | Brazil |
| NLD | Netherlands |
| TUR | Turkey |
| CAN | Canada |
| SWE | Sweden |
| MEX | Mexico |
| CHE | Switzerland |
| IND | India |
| PER | Peru |
| IDN | Indonesia |
| ECU | Ecuador |
| PHL | Philippines |
| DZA | Algeria |
| JPN | Japan |
| EGY | Egypt |
| PAK | Pakistan |
| DOM | Dominican Republic |
| COL | Colombia |
| KOR | South Korea |
| CHL | Chile |
| MAR | Morocco |
| ZAF | South Africa |
| AUS | Australia |
| CMR | Cameroon |
| BFA | Burkina Faso |
| NGA | Nigeria |
| TUN | Tunisia |
| COD | Democratic Republic of the Congo |
| NZL | New Zealand |
| GUM | Guam |
| MNP | Northern Mariana Islands |

**Note:** *These abbreviations* *were used in figure 3(e)* *for analysis on Death Rate distribution of COVID-19 of top 40 countries over the continent*
